# Potential Effects of Nicotinamide on Serum HDL-Cholesterol Levels and Hepatic Oxidative Stress, ABCA1 Gene and Protein Expression in Rats Fed a High-Fat/Fructose Diet

**DOI:** 10.3390/nu17213458

**Published:** 2025-11-01

**Authors:** Jesús I. Serafín-Fabián, Armando Ramírez-Cruz, J. D. Villeda-González, Jaime Gómez-Zamudio, Adrián Hernández-Díazcouder, Clara Ortega-Camarillo, Eugenia Flores-Alfaro, Miguel Cruz, Miguel Vazquez-Moreno

**Affiliations:** 1 Doctorado en Ciencias Biomédicas, Facultad de Ciencias Químico-Biológicas, Universidad Autónoma de Guerrero, Chilpancingo C.P. 39087, Mexico; fabianisimar@gmail.com; 2 Unidad de Investigación Médica en Bioquímica, Hospital de Especialidades, Centro Médico Nacional Siglo XXI, Instituto Mexicano del Seguro Social, Mexico City C.P. 06720, Mexico; ramirez@xanum.uam.mx (A.R.-C.); jaime.gomezz@imss.gob.mx (J.G.-Z.); adrian.hernandezdc@imss.gob.mx (A.H.-D.); clara.ortegac@imss.gob.mx (C.O.-C.); miguel.cruzlo@imss.gob.mx (M.C.); 3 Instituto de Fisiología Celular, Universidad Autónoma de Mexico, Mexico City C.P. 04510, Mexico; jvilleda@ifc.unam.mx; 4 Laboratorio de Investigación en Epidemiología Clínica y Molecular, Facultad de Ciencias Químico Biolóogicas, Universidad Autónoma de Guerrero, Chilpancingo C.P. 39087, Mexico; eugeniaflores@uagro.mx

**Keywords:** high-fat diet, high-fructose diet, nicotinamide, HDL, oxidative stress, ABCA1

## Abstract

A hypercaloric diet is associated with oxidative stress and the dysfunction of ATP-Binding Cassette transporter A1 (ABCA1), a key element in high-density lipoprotein (HDL) biogenesis and reverse cholesterol transport. Nicotinamide (NAM) presents antioxidant properties, which may contribute to maintaining lipid metabolism. Therefore, we aimed to evaluate the effect of NAM on HDL-cholesterol (HDL-C) level, oxidative stress markers, and the gene expression and protein levels of ABCA1 in Sprague-Dawley rats fed a hypercaloric diet. Forty male rats were divided into five groups: one group received a standard diet, and the remaining groups received a single high-fat, high-fructose diet (HFDF). Three of the HFDF groups received NAM treatment (5, 10, and 15 mM) in drinking water for 16 weeks (5 h/day). While HDL-C and oxidative stress were measured in serum samples, oxidative stress markers, and the gene expression and protein levels of ABCA1 were quantified in liver samples. The HDL-C level altered by the HFDF was improved by treatment with NAM. Furthermore, NAM reduces systemic lipid peroxidation levels and enhances the hepatic antioxidant response affected by the HFDF. In addition, NAM modulates the hepatic ABCA1 gene expression and protein level, altered by the HFDF. Our results suggest that NAM may modify the serum HDL-C level by an improvement of antioxidant response, and a possible modulation of the hepatic ABCA1 gene and protein expression. Further metabolic and molecular studies are needed to support the potential therapeutic role of NAM to prevent or treat lipid alterations promoted by a hypercaloric diet.

## 1. Introduction

The high intake of foods characterized by elevated fat and sugar content represents a global concern [1], due to its link with a significant burden of metabolic complications (including obesity, dyslipidemias, insulin resistance, type 2 diabetes, and cardiovascular diseases) considered as the leading causes of mortality and disability worldwide [2,3,4].

Increased consumption of a hypercaloric diet promotes the metabolic deregulation characterized by a low-grade chronic inflammation, oxidative stress, and dysregulation of the expression of genes involved in glucose and lipid metabolism [5,6]. In this context, the ATP-Binding Cassette transporter A1 (ABCA1) plays a central role in lipid homeostasis, as it participates in high-density lipoprotein (HDL) biogenesis and reverse cholesterol transport by facilitating the efflux of cholesterol from the intracellular to the extracellular compartment [7]. ABCA1 is in all tissues, and its expression is regulated by the nuclear receptors Liver X Receptors (LXR) [8]. Two isoforms of LXR have been identified, α and β, which share 77% sequence homology. The α isoform is predominantly expressed in macrophages, hepatocytes, and adipocytes, whereas the β isoform is expressed in all cell types [9].

Although it has been described that a high-calorie diet alters HDL levels in plasma, the mechanism that explains this deregulation has not been fully elucidated [10]. Experimental evidence suggests that a high fructose intake reduces hepatic *Lxr* and *Abca1* expression [11]. Similarly, it has been described that fat-rich diets disrupt lipid homeostasis through a reduction in *ABCA1* expression [12]. Both diets high in fructose and fat have been linked to increased generation of reactive oxygen species via dysfunctional mitochondria and overactivation of NADPH oxidase [13,14]. In this context, oxidative stress contributes to low LXR expression and reduced ABCA1 function, which consequently results in impaired cholesterol efflux and HDL biogenesis [15].

ABCA1 plays a key role in lipid homeostasis, highlighting the need to identify molecules capable of improving its expression in situations that compromise its function, such as the global dietary patterns that currently prevail [16]. In this regard, it has been described that nicotinamide (NAM), the amide form of niacin, presents antioxidant and anti-inflammatory properties. Regarding the antioxidant effect, it has been shown that NAM increases the concentration of glutathione in its reduced form (GSH), which enhances the antioxidant response [17,18]. On the other hand, the anti-inflammatory effect of NAM has been attributed to the decrease in TNF-α levels, which contributes to reducing the negative effects of inflammation [17,18]. In this context, NAM treatment, by enhancing the antioxidant response, could prevent the reduction in ABCA1 expression related to oxidative stress, which could improve HDL-C levels [19,20]. However, the effect of NAM on ABCA1 and HDL-C levels in relation to oxidative stress promoted by hypercaloric diets is currently unknown. Therefore, the present study aims to evaluate the effects of NAM supplementation on the high-density lipoprotein cholesterol (HDL-C) level, oxidative stress markers, and the hepatic gene and protein expression of LXR and ABCA1 in Sprague-Dawley rats fed a hypercaloric diet.

## 2. Materials and Methods

### 2.1. Animal Model

Male Sprague-Dawley rats with an average body weight of 250 ± 5 g (8 weeks old) were provided by the Animal Facility of the Centro Médico Nacional Siglo XXI of the Instituto Mexicano del Seguro Social (IMSS), and maintained under controlled conditions of humidity (50%) and temperature (23 °C). One week before the start of the experiments, rats were acclimated to light/dark cycles (12/12 h) and ventilation. The experimental design and animal management protocols were approved by the Local Committee of Research and Ethics in Health No. 3601 in the IMSS (CONBIOETICA-09-CEI-023-2017082 Approval date: 12 October 2023; Registration number R-2023-3601-233) and were conducted in compliance with the Norma Oficial Mexicana for the Care and Use of Laboratory Animals (NOM-062-ZOO-1999, revised 2015).

### 2.2. Experimental Design

**Figure 1:**
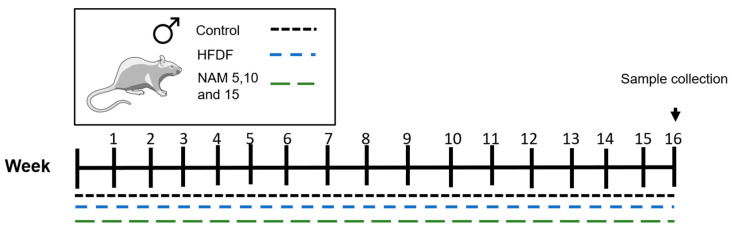
Animal model treatments with a hypercaloric diet and nicotinamide (NAM). Model: Control group, standard diet; HFDF group, high-fat diet + 40% fructose, and NAM 5, 10, and 15 groups, HFDF + 5, 10, and 15 mM NAM.

Animals were individually housed in acrylic cages and randomly assigned into five groups (*n* = 8 per group): (1) control; (2) high-fat diet + 40% fructose in drinking water (HFDF); (3) NAM 5; (4) NAM 10; (5) NAM 15. During 16 weeks, the control group received a standard diet (LABDIET 5008: 6.5% fat, 23% protein, and 46.5% carbohydrates) with tap water. The remaining groups were fed a non-commercial high-fat diet previously described by Ramírez-Cruz et al. [21] (18.5% fat, 15% protein, and 41.5% carbohydrates) with 40% fructose in drinking water (detailed diet composition in Supplementary Table S1). The groups NAM 5, NAM 10, and NAM 15 were provided with carbohydrate-free drinking water containing different concentrations of NAM (Sigma, catalog number N0636-100G): 5, 10, and 15 mM, respectively. NAM was prepared in drinking water before being administered in a separate water bottle for 5 h each morning (Figure 1). During treatment, food and liquid intake, as well as body weight, were monitored three times per week.

### 2.3. Sample Collection

At the end of the experimental treatments, animals, fasted for 10 h, were weighed and anesthetized with a combination of Zoletil (20 mg/kg, i.m.) and xylazine (5 mg/kg, i.m.). Blood samples were collected by intra-abdominal puncture of the inferior vena cava into tubes without an anticoagulant. Samples were centrifuged at 2000× *g* for 15 min. The resulting serum was stored at −70 °C until use. The liver was excised, dissected, and preserved at −70 °C until further analysis.

### 2.4. Serum Lipid Measurements

The concentrations of total cholesterol (TC), HDL-C, and triacylglycerols (TG) were determined by colorimetric enzymatic methods, using the COBAS Icobas 8000 modular analyzer (Hoffman-La Roche, Basel, Switzerland). The concentration of low-density lipoprotein cholesterol (LDL-C) and very low-density lipoprotein cholesterol (VLDL-C) was determined using the Friedewald formula [22].

### 2.5. Serum and Liver Lipid Peroxidation Measurements

Lipid peroxidation was assessed in serum samples and liver homogenates (prepared in 0.1 M PBS, pH 7.5) using the thiobarbituric acid reactive substances (TBARS) assay [23]. Briefly, 10% trichloroacetic acid was added to each aliquot of serum and liver homogenate. The mixtures were kept on ice and subsequently centrifuged at 20,000× *g* at 4 °C for 15 min. Thiobarbituric acid (0.11 mM) was then added to the resulting supernatants, which were vortexed and heated at 95 °C for 60 min. After cooling to room temperature, samples were vortexed again and centrifuged at 15,000× *g* at 4 °C for 15 min. The absorbance of the supernatants was measured at 535 nm. A reference standard was prepared using 1,1,3,3-tetramethoxypropane (malondialdehyde bis [dimethyl acetal]).

### 2.6. Serum and Liver GSH/GSSG Measurements

GSH concentrations were measured in liver tissue (10% *w*/*v*) homogenized in 5% metaphosphoric acid. The homogenates were centrifuged at 20,000× *g* at 4 °C for 20 min, and the resulting supernatants were collected. GSH levels in serum and supernatants were quantified spectrophotometrically using Ellman’s reagent [5,5′-dithiobis-(2-nitrobenzoic acid), DTNB]. This assay is based on the stoichiometric reaction between DTNB and the thiol group of GSH. The resulting product, nitro-mercaptobenzoate, exhibits a maximum absorbance at 412 nm, which is directly proportional to the GSH concentration (Tietze, 1969) [24]. For the determination of GSSG in liver tissue, it was homogenized (10% *w*/*v*) in 0.1 M PBS solution (pH 7.5), centrifuged at 10,000× *g* at 4 °C for 20 min, and the supernatants were collected for the analysis. For the GSSG assay, an enzymatic method was used after treating the supernatant and serum with 1-methyl-2-vinylpyridinium trifluoromethanesulfonate (10 mM), which binds the thiol groups in the samples without interfering with the glutaredoxin (GR) present in the assay. Subsequently, GR reduced GSSG to GSH in the presence of NADPH+, and the resulting GSH was quantified by reaction with Ellman’s reagent.

### 2.7. Analysis of Hepatic Lxr-α/β and Abca1 Gene Expression by RT-qPCR

Total RNA was isolated using TRIZOL reagent (Invitrogen, Carlsbad, CA, USA., cat. 15596018) following the manufacturer’s instructions, including extraction with chloroform and precipitation with isopropanol. RNA was resuspended in nuclease-free water, and its concentration and purity were determined by spectrophotometry. RNA integrity was evaluated in 1.5% agarose gels. For each sample, 2 µg of total RNA was used for reverse transcription to cDNA with the RevertAid Reverse Transcription Kit (Thermo Fisher Scientific, Waltham, MA, USA), according to the manufacturer’s protocol. Once cDNA was prepared, it was added to the reaction mixture to initiate qRT-PCR.

Quantitative real-time PCR was performed using a 7900HT Fast Real-Time PCR System (Applied Biosystems, Foster City, CA, USA). Reactions were carried out in a total volume of 5 µL, using SYBR Master Mix with 1 µL of forward primer and 1 µL of reverse primer. PCR conditions were: 95 °C for 10 min, followed by 40 cycles of 95 °C for 15 s and 60 °C for 60 s, then 95 °C for 10 s and 60 °C for 5 s. The 2^−ΔΔCT^ method was used to calculate mRNA expression levels, with *Rplp0* as the reference gene. Primer sequences for qRT-PCR are listed in Table 1.

### 2.8. Analysis of Hepatic LXR and ABCA1 Protein by Western Blot

Proteins were extracted using liver homogenates (10% *w*/*v*) with RIPA buffer (sc-24948A, Santa Cruz Biotechnology, Santa Cruz, CA, USA) following the manufacturer’s instructions. Protein concentrations were determined, and 30 µg of protein per sample was loaded for Western Blot analysis. Proteins were separated on 10% SDS-PAGE gels and subsequently transferred to PVDF membranes (IPVH00010, Millipore, Burlington, MA, USA). Membranes were blocked in 5% milk in TBS-Tween (TBST) for 2 h and incubated overnight with primary antibodies against ABCA1 (ab66217, Abcam, Cambridge, England), LXR (sc-377260, Santa Cruz Biotechnology, Santa Cruz, CA, USA), and actin (sc-8432, Santa Cruz Biotechnology, Santa Cruz, CA, USA). After incubation, membranes were washed with TBST and incubated at room temperature for 2 h with horseradish peroxidase-conjugated secondary antibody (anti-mouse, 1:10,000). Chemiluminescence was detected using a Kodak Gel Logic 200 imaging system/scanner (Rochester, New York, NY, USA). The densitometric analysis was performed using ImageJ software (version 1.54f, National Institutes of Health, Bethesda, MD, USA; available at URL https://imagej.net/ij/ accessed 3 October 2025).

### 2.9. Data Analysis

Results are presented as mean ± standard error of the mean (SEM). Comparisons between groups were performed using one-way ANOVA, followed by post hoc analysis with Dunnett’s test (HFDF: reference group). All statistical analyses were conducted using GraphPad Prism 8 software, and *p*-values < 0.05 were considered statistically significant.

## 3. Results

### 3.1. Body Weight and Energy Intake in Rats Fed a Hypercaloric Diet

Animals were monitored throughout the 16-week study period, during which food, liquid, fructose, and energy intake, as well as weight gain, were analyzed (Table 2). Although the HFDF consumed a significantly lower amount of food compared with the control group, its total energy intake was higher. Regarding liquid and body weight gain, no significant differences were observed between the control, NAM 5, NAM 10, and NAM 15 groups compared to the HFDF group.

### 3.2. Effect of Nicotinamide on Serum Lipid Profile in Rats Fed a Hypercaloric Diet

The serum lipid profiles of the control, HFDF, and NAM treatment groups are presented in Table 3. Compared to the control, the HFDF group exhibited significantly higher levels of TG, TC, LDL-C, and VLDL-C, along with a reduced HDL-C concentration. In comparison with the HFDF, the three NAM groups showed reduced levels of TC. For TG and VLDL-C, the NAM 5 and NAM 10 groups presented lower levels than HFDF. In the case of LDL-C, the NAM 15 group showed significantly lower concentration than HFDF. HDL-C levels were significantly increased in the NAM 10 group compared with the HFDF.

### 3.3. Effect of Nicotinamide on Lipid Peroxidation Level and Redox Balance (GSH/GSSG) in Serum and Liver Samples of Rats Fed a Hypercaloric Diet

**Figure 2:**
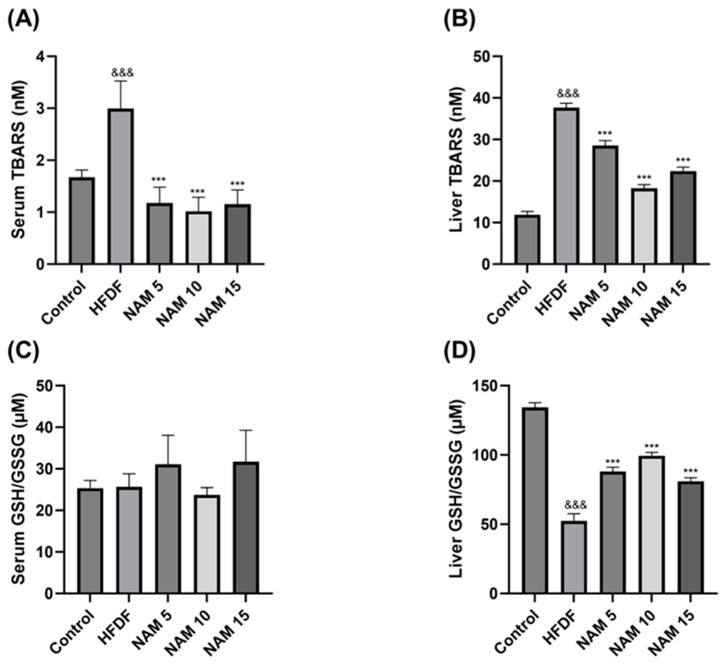
Effect of nicotinamide on lipid peroxidation level and redox balance (GSH/GSSG) in serum and liver samples. (**A**,**B**) TBARS levels in serum and liver samples. (**C**,**D**) GSH/GSSG levels in serum and liver samples. The groups are: Control (standard diet), HFDF (High Fat, High Fructose Diet), and HFDF treated with NAM at doses of 5, 10, and 15 mM. Data are presented as mean ± standard error of the mean. *n* = 8 per group. Abbreviation: TBARS; Thiobarbituric acid reactive substances, Ratio GSH/GSSG; Ratio reduced glutathione/oxidized glutathione. Statistical analysis was performed by one-way ANOVA followed by Dunnett’s post hoc test. Significant differences are denoted as follows: ampersands (&) indicate comparison with the control group (^&&&^ *p* < 0.001), and asterisks (*) indicate comparison with the HFDF group (*** *p* < 0.001).

The levels of lipid peroxidation in serum and liver samples of the control, HFDF, and NAM-treated groups are represented in Figure 2A,B. Compared to the control, the HFDF group exhibits significantly higher TBARS levels in serum and liver samples. Treatments with NAM 5, 10, and 15 mM presented significantly lower TBARS levels in serum and liver samples than the HFDF group.

The GSH/GSSG ratio in serum and liver samples of the control, HFDF, and NAM-treated groups is shown in Figure 2C,D. In the liver, the GSH/GSSG ratio was significantly decreased in the HFDF compared to the control group. Compared with the HFDF group, treatments with NAM 5, 10, and 15 mM significantly increased the GSH/GSSG ratio in the liver. No significant differences were observed in serum among any groups.

### 3.4. Effect of Nicotinamide on the Gene Expression and Protein Levels of LXR and ABCA1 in Liver Samples of Rats Fed a Hypercaloric Diet

**Figure 3:**
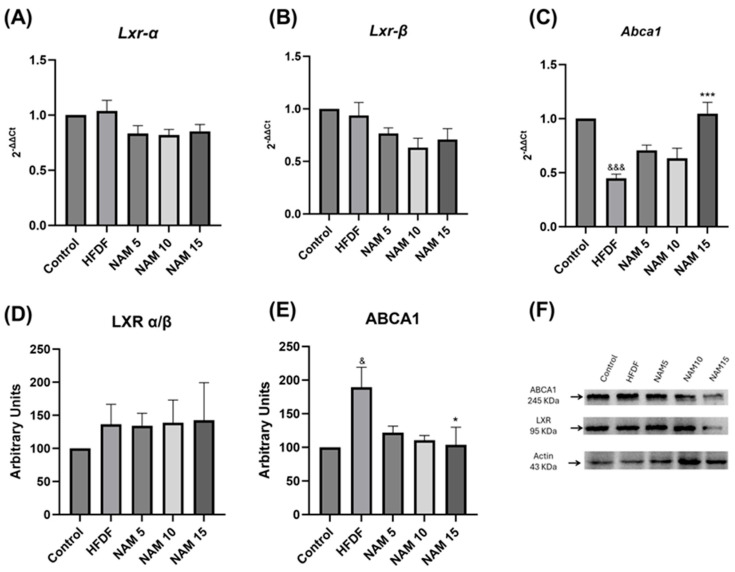
Gene expression and protein levels of LXR and ABCA1 in hepatic tissue of rats fed a hypercaloric diet and treated with nicotinamide at 5, 10, and 15 mM. (**A**) *Lxr-α* RNA expression, (**B**) *Lxr-β* RNA expression, (**C**) *Abca1* RNA expression, (**D**) LXR α/β protein levels, (**E**) ABCA1 protein levels and (**F**) Western blot representative image. The groups are: Control (standard diet), HFDF (High Fat, High Fructose Diet), and HFDF treated with NAM at doses of 5, 10, and 15 mM. Data are presented as mean ± standard deviation. *n* = 6 per group. Data are expressed as mean ± standard error. Statistical analysis was performed by one-way ANOVA followed by Dunnett’s post hoc test. Significant differences are denoted as follows: ampersands (&) indicate comparison with the control group (^&^ *p* < 0.05; ^&&&^ *p* < 0.001), and asterisks (*) indicate comparison with the HFDF group (* *p* < 0.05; *** *p* < 0.001).

The gene expression of *Lxr* and *Abca1* in liver samples of control, HFDF, and NAM-treated groups is represented in Figure 3A–C. No significant differences were observed in *Lxr α/β* gene expression (Figure 3A,B). However, HFDF exhibits lower *Abca1* gene expression compared with the control group (Figure 3C). Additionally, the NAM 15 group showed significantly higher *Abca1* gene expression than the HFDF group (Figure 3C).

LXR α/β and ABCA1 protein levels in liver samples of control, HFDF, and NAM-treated groups are shown in Figure 3D–F. Comparison of LXR α/β protein levels exhibited no significant differences among all groups (Figure 3D,F). Nevertheless, ABCA1 protein level was significantly higher in the HFDF compared to the control group, whereas treatment with NAM 15 mM reduced ABCA1 protein level relative to the HFDF group (Figure 3E,F).

## 4. Discussion

High-calorie diets are currently considered a global problem because they are related to the increase in incidence and morbidity of metabolic diseases that most impact the social and economic systems worldwide [1,2]. In the present study, the effect of NAM on HDL-C level and oxidative stress markers in serum, as well as the hepatic gene expression and protein levels of LXR and ABCA1 of Sprague-Dawley rats fed a hypercaloric diet, was evaluated.

Although the HFDF groups consumed less food than the control group, their total energy intake was higher due to the caloric density of the diet. However, no significant differences in body weight gain were observed between groups. This finding is consistent with previous studies suggesting that the lack of data showing body weight gain could be related to a loss of muscle tissue and gain of adipose tissue [25]. Other possible factors that could be attributed to the lack of difference in body weight gain are the short duration of the model, age of the animals, and the caloric composition of the diet (lipid, carbohydrate, and protein content) [26,27,28]. However, these studies on dietary models without changes in body weight gain also evidence a significant metabolic effect related to lipid metabolism and even impaired liver integrity.

The HFDF group exhibited significantly higher levels of TC, LDL-C, TG, and VLDL-C, and decreased concentration of HDL-C. In contrast, NAM-treated groups reduced TG, TC, LDL, and VLDL levels and increased HDL-C concentrations, compared to the HFDF group, which is consistent with previous reports for NAD precursors, such as niacin and nicotinamide riboside. This effect has been attributed to the inhibition of hepatic diacylglycerol acyltransferase 2 (DGAT2) [29], an enzyme involved in TG synthesis. Furthermore, niacin has been shown to reduce intracellular apoB levels and, consequently, the formation of LDL-C [30]. Notably, HDL-C was significantly increased in the treatment with NAM 10 mM compared to the HFDF group. This result is similar to the effects that niacin treatment has demonstrated in epidemiological studies [31]. Considering that a high-fat and fructose diet affects HDL-C levels through the deregulation of *Abca1* expression and function, our results suggest that NAM may be a molecule capable of improving lipid homeostasis related to the hepatic ABCA1 transporter.

Our results evidenced the prooxidant effect of the lipid and fructose diet, which has been reported in previous studies [32,33]. The HFDF group had higher serum and hepatic levels of TBARS and a reduced hepatic GSH/GSSG ratio than the control group. However, we also demonstrate the antioxidant effect of NAM. In all three NAM treatment groups, serum and hepatic TBARS levels were significantly reduced compared to the HFDF group. Additionally, the three NAM treatment groups showed the restoration of the GSH/GSSG ratio in hepatic samples of the HFDF group. These results are consistent with previous studies that show the antioxidant effect of NAM in the presence of oxidative stress induced by different diets, particularly with fructose, or in combination with fat and disaccharides [18,21,34]. The antioxidant effect of NAM in the context of diet-induced oxidative stress may be explained through its contribution to the nicotinamide adenine dinucleotide phosphate (NADPH), a key molecule in the glutathione reductase via to recycle GSH from GSSG, which enhances the antioxidant capacity [34,35]. Serum GSH/GSSG levels did not show significant differences, which may be attributed to the primarily intracellular role of GSH that is not readily reflected in a heterogeneous sample such as serum [36,37].

A high-fat and fructose diet is known to disrupt lipid homeostasis and affect key regulatory proteins such as ABCA1. Therefore, we evaluated the gene expression of *Lxr* and *Abca1* in the control, HFDF, and NAM treatment groups. No significant changes were observed in either *Lxr* gene expression isoforms among the groups. However, *Abca1* gene expression was significantly reduced in the HFDF compared to the control group. Although the full mechanism is not completely elucidated, it has been described that the ABCA1 transcription may be regulated through oxidative stress and the inflammation process, without activating LXR [38]. Additionally, in comparison with HFDF, increased *Abca1* gene expression was observed in the NAM 15 group, which is consistent with the effect of niacin on *Abca1* gene expression, suggesting that NAM may exert a similar regulatory effect [18]. This finding may be explained by the key role of NAM in the production of nicotinamide adenine dinucleotide (NAD+), an important cofactor in redox reactions, which promotes enhanced expression of genes involved in mitochondrial biogenesis and the regulation of cholesterol metabolism [18,39,40]. In this context, sirtuins have been associated with the regulation of genes involved in antioxidant defense and lipid metabolism, improving ABCA1 activity and HDL-C levels [41].

The effect of NAM on the LXR α/β and ABCA1 protein levels was also evaluated. The LXR protein level was consistent with *LXR α/β* gene expression, showing no significant changes among the groups. In contrast, the ABCA1 protein level exhibited an inverse pattern with our result regarding *ABCA1* gene expression. The HFDF group showed significantly higher ABCA1 protein level compared to the control, which is in line with previous studies with high-fat diets [42]. It has been suggested that fructose-induced oxidative damage promotes increased levels of miR-33, a microRNA that has also been related to decreased *ABCA1* gene expression [43,44]. In this context, it is possible that the antioxidant effect of NAM may explain the higher *Abca1* gene expression in the NAM 15 nM treatment group. Interestingly, treatments with NAM 5 and 10 nM decreased ABCA1 protein level compared to the HFDF group, suggesting that, independently of gene expression, NAM may be involved in the post-translational regulation of ABCA1. The discrepancy between the protein level and the expression of the *Abca1* gene has been previously reported [45], and it has been proposed that this fact may be related to post-translational modifications, which could enhance the half-life and functional activity of the ABCA1 protein [8,46].

As a major strength of this study, it is important to highlight the integration of metabolic, redox balance, and molecular markers related to ABCA1 activity in the presence of NAM as a regulatory agent of the negative effects of the hypercaloric diet. Our results evidence a negative impact of the HFDF diet on lipid metabolism, as well as established oxidative stress in the liver. Furthermore, the observed improvements in HDL-C level, combined with restoration of the GSH/GSSG ratio and increased *Abca1* gene expression in the liver of NAM-treated groups, highlight its potential antioxidant effect. These evidences encourage the development of complementary biochemical and molecular research to elucidate the mechanism of action of NAM to propose it as a possible therapeutic molecule to prevent or treat dyslipidemia and oxidative stress generated by high-calorie diets.

Our study also has some limitations. For example, the selected diet was based on common food consumption patterns of the global population, which prevents individual assessment of the effects of a high-fat or high-fructose diet alone. Furthermore, we did not analyze NAD+ levels or specific metabolites related to NAM metabolism. We also did not consider the potential involvement of other molecules that may contribute to the regulation of *Lxr* and *Abca1* gene expression (e.g., inflammatory markers), which could have revealed other potential pathways through which NAM exerts its beneficial effects. Finally, our experimental design is relatively general, aiming to target an important regulator of the most widely evaluated serum lipid profile in clinical practice for cardiovascular risk stratification: CT, TG, cLDL, cVLDL, and cHDL. Even with these limitations, our study expands the understanding of lipid metabolism and strengthens the evidence to justify future research seeking to contribute to the prevention and treatment of lipid metabolism complications related to high-calorie diets.

## 5. Conclusions

Our results suggest that NAM treatment may modulate the serum HDL-C level by an improvement of the hepatic antioxidant response, and a possible modulation of the hepatic ABCA1 gene and protein expression. These evidences encourage the development of complementary metabolic and molecular research to elucidate the mechanism of action of NAM to propose it as a possible therapeutic molecule to prevent or treat dyslipidemia and oxidative stress generated by high-calorie diets.

## Figures and Tables

**Table 1:** Specific primer sequences for target genes.

Genes	Gen ID	Forward Primer	Reverse Primer
*Lxr-α*	58852	AGTCACGCCTTGGCCCATTGC	CGGACACGATGGCCAGCTCA
*Lxr-β*	58851	GGCCGGGAGGACCAGAT	GCGTCTGGCTGTCTCTAGCAA
*Abca1*	313210	ACCGACAAGGCCGCACCATT	GCCCACACAACACAGCTTCCCA
*Rplp0*	64205	AGCCAAGGTCGAAGCAAA	GCTTAGTCGAAGAGACCGAATC

**Table 2:** Zoometric data in rat groups.

Group	Control	HFDF	NAM 5	NAM 10	NAM 15
	(*n* = 8)	(*n* = 8)	(*n* = 8)	(*n* = 8)	(*n* = 8)
Food	24.470 ± 0.476	16.490 ± 0.415 ^&&&^	16.920 ± 0.640	16.530 ± 0.482	15.100 ± 0.554
(g/day/rat)					
Liquid	32.480 ± 1.637	35.400 ± 1.934	29.960 ± 1.227	30.140 ± 1.583	30.540 ± 1.624
(mL/day/rat)					
Fructose	-	8.922 ± 0.951	7.903 ± 0.684	9.070 ± 0.647	9.264 ± 0.657
(g/day/rat)					
Energy	91.640 ± 1.783	114.500 ± 4.126 ^&&^	110.500 ± 4.629	112.900 ± 4.754	106.900 ± 5.027
(Kcal/day/rat)					
Body Weight Gain (g)	238.000 ± 10.090	210.800 ± 6.346	202.300 ± 7.365	212.100 ± 8.346	215.100 ± 6.634

The groups are: Control (standard diet), HFDF (High Fat, High Fructose Diet), and HFDF treated with NAM at doses of 5, 10, and 15 mM. Data are expressed as mean ± standard error of the mean. One-way ANOVA followed by Dunnett’s post hoc test was used for statistical analysis. Significant differences are denoted as follows: ampersands (&) indicate comparison with the control group (^&&^ *p* < 0.01; ^&&&^ *p* < 0.001).

**Table 3:** Serum lipid levels in rats fed a hypercaloric diet and treated with nicotinamide at 5, 10, and 15 mM.

Group	Control	HFDF	NAM 5	NAM 10	NAM 15
	(*n* = 8)	(*n* = 8)	(*n* = 8)	(*n* = 8)	(*n* = 8)
TG	105.805 ± 11.236	225.816 ± 21.322 ^&&^	121.211 ± 21.114 **	121.152 ± 19.024 *	159.510 ± 20.355
(mg/dL)					
TC	63.751 ± 0.818	75.886 ± 1.469 ^&&&^	66.002 ± 1.500 *	68.883 ± 1.246 *	62.002 ± 1.964 ***
(mg/dL)					
LDL-C	12.451 ± 0.457	17.863 ± 1.085 ^&^	13.995 ± 1.645	16.281 ± 1.430	8.950 ± 0.580 **
mg/dL					
VLDL-C	17.248 ± 1.333	38.153 ± 3.123 ^&&^	20.801 ± 3.261 **	18.281 ± 2.506 **	30.207 ± 3.493
mg/dL					
HDL-C	33.254 ± 2.206	25.651 ± 1.346 ^&^	31.195 ± 2.416	34.534 ± 1.668 *	31.982 ± 1.823
mg/dL					

The groups are: Control (standard diet), HFDF (High Fat, High Fructose Diet), and HFDF treated with NAM at doses of 5, 10, and 15 mM. Data are expressed as mean ± standard error of the mean. Abbreviations: TC, total cholesterol; LDL-C, low-density lipoprotein cholesterol; HDL-C, high-density lipoprotein cholesterol; VLDL-C, very-low-density lipoprotein cholesterol; TG, triacylglycerols. Statistical analysis was performed by one-way ANOVA followed by Dunnett’s post hoc test. Significant differences are denoted as follows: ampersands (&) indicate comparison with the control group (^&^ *p* < 0.05; ^&&^ *p* < 0.01; ^&&&^ *p* < 0.001), and asterisks (*) indicate comparison with the HFDF group (* *p* <0.05; ** *p* < 0.01, *** *p* < 0.001).

## Data Availability

The dataset generated during and/or analyzed during the current study is available from the corresponding author on reasonable request.

## Supplementary Materials

The following supporting information can be downloaded at: https://www.mdpi.com/article/10.3390/nu17213458/s1, Table S1: The composition of normal chow and high fat diet.

## Abbreviations

The following abbreviations are used in this manuscript:

ABCA1	ATP-Binding Cassette Transporter A1 (protein)
*Abca1*	ATP-Binding Cassette Transporter A1 (gene)
HDL	High-density lipoprotein
NAM	Nicotinamide
HFDF	High-fat, high-fructose diet
LXR	Liver X Receptor (protein)
*Lxr*	Liver X Receptor (gene)
IMSS	Instituto Mexicano del Seguro Social
TC	Total cholesterol
LDL-C	Low-density lipoprotein cholesterol
HDL-C	High-density lipoprotein cholesterol
VLDL	Very low-density lipoprotein cholesterol
TG	Triacylglycerols
TBARS	Thiobarbituric acid reactive substances
GSH	Reduced glutathione
GSSG	Oxidized glutathione
GSH/GSSG	Ratio reduced glutathione/oxidized glutathione
DGAT2	Diacylglycerol acyltransferase 2
NAD+	Nicotinamide adenine dinucleotide
