# Supplementary material for: Potential Effects of Nicotinamide on Serum HDL-Cholesterol Levels and Hepatic Oxidative Stress, ABCA1 Gene and Protein Expression in Rats Fed a High-Fat/Fructose Diet
Source: Nutrients. 2025 Nov 1;17(21):3458. doi: 10.3390/nu17213458 (PMC12610202; doi:10.3390/nu17213458)
Supplement: Supplementary file 1 [file nutrients-17-03458-s001.zip › nutrients-3948607-supplementary.pdf]

Supplementary Table S1. The composition of normal chow and high fat diet

	**LABDIET 5008**	**High Fat Diet**
**Protein**	23%	11.76%
**Fats**	**6.50%**	**18.38%**
**Fiber**	4%	19.52%
**Ashes**	8%	3.59%
**Minerals**	2.50%	2.50%
**Carbohydrates**	56%	44.25%
